# Surgical and Injection Interventions for Dupuytren's Disease: A Systematic Review Protocol

**DOI:** 10.1055/s-0045-1811595

**Published:** 2025-09-08

**Authors:** Rodrigo Guerra Sabongi, Vinicius Ynoe de Moraes, João Baptista Gomes dos Santos, Vinicius Tassoni Civile, Nelson Carvas Junior, Flávio Faloppa

**Affiliations:** 1 Disciplina de Cirurgia da Mão e Membro Superior, Escola Paulista de Medicina, Universidade Federal de São Paulo, São Paulo, SP, Brazil; 2 Programa de Pós-graduação em Saúde Baseada em Evidência, Universidade Federal de São Paulo, São Paulo, SP, Brazil

**Keywords:** collagenases, Dupuytren contracture, fasciotomy, systematic review', colagenases, contratura de Dupuytren, fasciotomia, revisão sistemática

## Abstract

**Objective:**

To compare the effectiveness and safety of surgical and injection-based interventions for Dupuytren's disease (DD) using systematic review and network meta-analysis methodology.

**Methods:**

The current protocol follows the Preferred Reporting Items for Systematic Reviews and Meta-Analyses Protocols (PRISMA-P) guidelines and is registered in The International Prospective Register of Systematic Reviews (PROSPERO). Randomized controlled trials involving adult patients with DD treated by surgical (e.g., fasciectomy, fasciotomy) or injection-based interventions (e.g., collagenase, corticosteroids) must be included. A comprehensive search must be conducted across MEDLINE, CENTRAL, EMBASE, LILACS, IBECS, and trial registries, without language or date restrictions. Two reviewers must perform independent screening, data extraction, and risk of bias assessment (RoB2). Primary outcomes must include functional improvement and adverse effects. Secondary outcomes must include recurrence, range of motion, and pain. Data synthesis should involve random-effects pairwise and network meta-analyses, with GRADE used to assess certainty of evidence. Subgroup analyses should explore heterogeneity based on clinical and methodological variables.

**Results:**

This proposal of review aims to generate comparative estimates of effectiveness and safety across all eligible interventions, incorporating both direct and indirect evidence. Functional outcomes must be synthesized using a predefined hierarchy of patient-reported outcome measures (PROMs), and treatments must be ranked based on efficacy and safety profiles.

**Conclusion:**

The present systematic review aims to fill current evidence gaps by comparing all relevant interventions for DD, supporting clinical decision-making through robust synthesis of functional and safety outcomes.

## Resumo

**Objetivo:**

Comparar a eficácia e a segurança de intervenções cirúrgicas e injetáveis para tratamento da contratura de Dupuytren (CD) por meio de revisão sistemática e metodologia de metanálise em rede.

**Métodos:**

Este protocolo segue as diretrizes *Preferred Reporting Items for Systematic Reviews and Meta-Analyses Protocols* (PRISMA-P) e está registrado no *The International Prospective Register of Systematic Reviews* (PROSPERO). Devem ser incluídosestudos clínicos randomizados com pacientes adultos com CD e submetidos a intervenções cirúrgicas (ex.: fasciectomia e fasciotomia) ou injetáveis (ex.: colagenase e corticosteroides). Uma busca abrangente deve ser conduzida nas bases de dados MEDLINE, CENTRAL, EMBASE, LILACS, IBECS e registros de estudos clínicos, sem restrições de idioma ou data. Dois revisores devem realizer a triagem, extração de dados e avaliação de risco de viés (RoB2) de forma independente. Os desfechos primários devem incluir melhora funcional e efeitos adversos. Os desfechos secundários devem incluir recidiva, amplitude de movimento e dor. A síntese de dados deve envolver metanálises pareadas e de rede de efeitos aleatórios e a certeza da evidência deve ser avaliada de acordo com a abordagem *Grading of Recommendation, Assessment, Development and Evaluation* (GRADE). As análises de subgrupos devem explorar a heterogeneidade com base em variáveis clínicas e metodológicas.

**Resultados:**

Esta proposta de revisão pretende gerar estimativas comparativas de eficácia e segurança em todas as intervenções elegíveis, incorporando evidências diretas e indiretas. Os desfechos funcionais devem ser sintetizados usando uma hierarquia predefinida de medidas de desfecho relatadas pelo paciente (PROMs, do inglês *patient-reported outcome measures* ) e os tratamentos devem ser classificados com base nos perfis de eficácia e segurança.

**Conclusão:**

Esta revisão sistemática visa preencher lacunas de evidências atuais comparando todas as intervenções relevantes para o tratamento da CD, apoiando a tomada de decisão clínica por meio de síntese robusta de resultados funcionais e de segurança.

## Introduction

Dupuytren's disease (DD) is a benign and progressive fibroproliferative disorder of the palmar fascia, characterized by the formation of nodules and cords that may evolve into flexion contractures, most frequently affecting the metacarpophalangeal (MCP) and proximal interphalangeal (PIP) joints. 1 2 The condition results from the abnormal proliferation and differentiation of fibroblasts into contractile myofibroblasts, leading to thickening, shortening, and loss of digits' extension. 1

Although its etiology is multifactorial, with strong genetic influence, several modifiable and nonmodifiable risk factors have been consistently associated with the disease, including increased age, male sex, alcohol consumption, smoking, diabetes mellitus, epilepsy, liver disease, and occupations involving manual labor or vibratory exposure. 3 The estimated prevalence in the general population ranges from 4 to 6%, with higher rates observed in individuals of Northern European ancestry and older age groups. 2 4 In advanced stages, the disease can significantly impair hand function and quality of life.

Treatment options for DD have evolved over centuries, with surgical interventions such as fasciotomy and fasciectomy being the primary approaches since the 17 ^th^ century. 5 In recent years, less invasive treatments have been increasingly explored, particularly intralesional ones that aim to modify disease progression in early stages or offer alternatives to surgery. Injectable collagenase *Clostridium histolyticum* (CCH), which enzymatically disrupts the collagen cords, has demonstrated short-term efficacy in reducing contractures, especially at the MCP joint, and became widely used after regulatory approval, although its availability has varied by region. 6 Other agents such as corticosteroids, injected into early nodules, have been associated with partial regression or disease stabilization, 7 while biologic therapies targeting molecular pathways, including anti-tumor necrosis factor (TNF) agents, are under investigation with promising early-phase results. 8

Among surgical techniques, percutaneous needle fasciotomy (PNF) represents a minimally invasive option associated with rapid recovery and low morbidity. However, it carries a high rate of recurrence, particularly in patients with aggressive disease. Limited fasciectomy remains the standard treatment for more advanced contractures, offering greater durability at the cost of longer recovery and higher complication rates. 5 8 In recurrent or severe cases, dermofasciectomy may be indicated, although it involves skin grafting and presents higher surgical complexity and morbidity. The choice of intervention depends on multiple factors including severity, joint involvement, comorbidities, recurrence risk, and surgeons' experience. 9

Despite the availability of various treatments for DD, there is a notable gap in the literature regarding comprehensive comparisons of these interventions. The lack of robust comparative syntheses limits the ability of clinicians to identify which interventions offer the best balance of effectiveness, safety, and long-term outcomes, particularly with respect to functional improvement, adverse effects, and recurrence rates.

Previous systematic reviews have typically focused on individual treatment modalities—such as collagenase, needle fasciotomy, or limited fasciectomy—excluding other relevant surgical techniques and injection therapies, such as corticosteroid or experimental biological agents. 10 11 12 13 14 Only one review conducted a network meta-analysis (NMA) and, even then, the comparison was restricted to three interventions. 15 Only one Cochrane review, published in 2015, attempted broader comparisons but included only surgical techniques and did not incorporate newer modalities. 16

Several recent reviews have also presented important methodological limitations that hinder their applicability in clinical decision-making. Many restricted inclusion to studies published in English or sourced only from traditional databases, potentially overlooking relevant data from other regions or languages. 10 11 15 Additionally, the selection of patient-reported outcome measures (PROMs) has been inconsistent, often lacking a clear hierarchy or justification. 11 12 13 Several recent reviews did not include NMA, thereby restricting their capacity to compare interventions beyond direct pairwise analyses. 10 11 12 13 14

Finally, most of the existing reviews are now methodologically outdated, having completed their literature searches more than 5 years ago, prior to the publication of several key randomized controlled trials.

Collectively, these limitations reinforce the need for an updated, comprehensive, and methodologically rigorous systematic review to support evidence-based clinical decision-making. This protocol outlines the methodology for a systematic review and NMA aiming to compare the effectiveness and safety of surgical and injection-based interventions for the treatment of DD.

## Materials and Methods

The present review was conducted according to the Preferred Reporting Items for Systematic Reviews and Meta-Analyses for Network Meta-Analyses (PRISMA-NMA) guidelines. 17 This study has been Registered on The International Prospective Register of Systematic Reviews (PROSPERO) database, under the number CRD42023439436. The Preferred Reporting Items for Systematic Reviews and Meta-Analyses Protocols (PRISMA-P) checklist was used for the development of this protocol. 18 Any substantial amendments will be documented and updated in the PROSPERO registry.

### Criteria for Considering Studies

#### Study Population and Study Design

The proposed review must include randomized controlled trials (RCTs) evaluating surgical and injection-based interventions for DD. Eligible studies must include participants aged 18-years or older, diagnosed with DD, and treated with either surgical or injection interventions. To ensure the assessment of long-term outcomes, only trials with a follow-up period longer than 3 months should be included. No restrictions must be applied regarding publication status or language, allowing the inclusion of both published and unpublished studies.

#### Definitions and Outcome Measures

The studies must include a comparison arm that includes infiltration and/or surgical treatment. Studies comparing the following interventions were eligible for inclusion in this review:

- Surgical interventions: total or partial fasciectomy; percutaneous fasciotomy, among others.
- Infiltration interventions: any treatments using active substances such as CCH or corticosteroids, among others.

The primary outcomes of the proposed review will focus on functional improvement and adverse effects. Functional improvement can be assessed using patient-reported scales, following a predefined hierarchy of instruments, from more to less disease-specific scales ( Table 1 ). 19 20 21 22 23 24

**Table 1:** Hierarchy of PROMs for assessing functional improvement in DD

Rank	Instrument	
1	SDSS 19	
2	DIF-CHUM 20	
3	URAM 21	
4	DASH and/or QuickDASH 22	
5	MHQ and/or briefMHQ 23	
6	CHFS 24	

**Abbreviations:** CHFS, Cochin Rheumatoid Arthritis Hand Function Scale; DASH, Disability of the Arm, Shoulder, and Hand Questionnaire; DD, Dupuytren's disease; DIF-CHUM, Dupuytren's Contracture Impact on Function - Centre Hospitalier de l'Université de Montréal; MHQ, Michigan Hand Questionnaire; PROM, patient-reported outcome measure; SDSS, Southampton Dupuytren's Scoring Scheme; URAM, Unité Rhumatologique des Affections de la Main.

Adverse effects should be categorized into minor and major complications and ranked by order of interest for analysis. Minor complications include superficial infection, wound dehiscence, delayed healing, and hematoma. Major complications consist of deep infection, the need for reoperation, skin coverage, ischemia, necrosis, nerve injury, tendon rupture, intensive care unit admission, and death. The following complications will be prioritized in the analysis, in descending order of interest: nerve injury, delayed healing, need for reoperation, superficial infection, and deep infection.

Secondary outcomes will include range of motion, measured through contracture angle, total range of motion, or Tubiana's stage. Pain will be assessed using the visual analog scale (VAS). Recurrence will be defined as more than 20 degrees of contracture in any treated joint 1-year after treatment, compared to 6 weeks posttreatment, with each treated joint evaluated individually. 25

#### Search Methods for Identification of Studies

Relevant studies should be identified by searching the electronic databases and clinical trial registries: Cochrane Library, Medline, Embase, Lilacs, Ibecs, ClinicalTrials.gov, and the World Health Organization International Clinical Trials Registry Platform. These searches will be conducted without language or publication date restrictions. The full search strategy for each database is provided in Appendix 1 . Additionally, manual searches should include reviewing reference lists from relevant conference abstracts and proceedings, and content experts and authors must be contacted to identify unpublished or ongoing studies.

### Data Collection and Analysis

#### Study Selection

The screening process should be conducted using the Rayyan (Qatar Foundation) software. Two authors must independently screen titles and abstracts to identify potentially eligible studies based on predefined eligibility criteria. Full-text articles should be retrieved for studies included in the initial screening, followed by assessment of their eligibility for final inclusion. In the event of disagreements, a third reviewer should be consulted to resolve conflicts. The selection process must be documented in a PRISMA flowchart, detailing reasons for excluding studies.

#### Analysis and Data Synthesis

Data extraction and management should be conducted independently by two authors using a standardized form in Microsoft Excel (Microsoft Corp.). Extracted data must include study methods (design, definition), identification data (sponsorship, country, author details), participant characteristics (number randomized, analyzed, and lost to follow-up), baseline data (age, sex, disease severity, body mass index [BMI], disease duration), interventions (description, group sizes), outcome measures (type, scale, unit), study design characteristics, and risk of bias assessment. Tables summarizing study characteristics and risk of bias must be created. For continuous outcomes, means, standard deviations (SD), and participant numbers should be extracted, with data transformation as needed. For dichotomous outcomes, event rates and participant numbers will be extracted. Intention-to-treat analysis should be applied whenever possible. Discrepancies must be resolved through consensus, and final data must be exported to RStudio (Posit Software, PBC) for analysis.

For continuous outcomes, means, SDs, and participant numbers should be extracted. If alternative measures of dispersion are reported (e.g., confidence intervals or standard errors), SDs must be be calculated according to the Cochrane Handbook. Standardized mean differences (SMD) must be used for outcomes reported on different scales, with results expressed as 95% confidence intervals (CIs). For dichotomous outcomes, event rates and participant numbers must be extracted to calculate odds ratios (ORs) with 95% CIs.

The unit of analysis considered is each participant. For studies reporting multiple time points, data from the longest follow-up must be extracted. For missing data, study authors should be contacted. If unavailable, the study must proceed with the available data, and sensitivity analyses will assess the potential impact. Intention-to-treat (ITT) analysis should be used whenever possible.

Heterogeneity must be be assessed based on participant characteristics, interventions, and outcomes. The statistical results should be be evaluated using forest plots and quantified with the I ^2^ statistic, and a Chi-squared test ( *p* < 0.1) to detect significance. Heterogeneity is to be classified as not important (0–40%), moderate (30–60%), substantial (50–74%), or considerable (75–100%). Subgroup analyses can be conducted to explore potential sources of heterogeneity.

Tables with a summary of findings should be prepared for all outcomes, using the most appropriate reference comparator. Certainty of evidence will be assessed using the Grading of Recommendations, Assessment, Development and Evaluation (GRADE) approach by two authors. Certainty must be downgraded based on factors such as study limitations, inconsistency, and publication bias, with results categorized as high, moderate, low, or very low. Disagreements should be resolved by consensus, with justifications for downgrades placed in the footnotes.

Subgroup analyses should investigate heterogeneity by age, comorbidities (e.g., diabetes, epilepsy, alcoholism, smoking), disease duration, severity (Tubiana stage/total range of motion), and surgical techniques (e.g., open palm, McCash, Bruner incision, skin graft, z-plasty). Sensitivity analyses should be conducted to test the robustness of findings, focusing on the inclusion of unpublished studies and those with a high risk of bias.

### Quality Assessment

The risk of bias for each included study must be independently assessed by two review authors using version 2 of the Risk of Bias (RoB2, Cochrane Collaboration) tool, following the recommendations in the Cochrane Handbook for Systematic Reviews of Interventions, version 6.3. Any disagreements must be resolved through consensus. The assessment should focus on bias across several domains: bias arising from the randomization process, deviations from intended interventions, missing outcome data, measurement of outcomes, and selection of reported results. For each domain, responses to signaling questions will result in a judgment of “low risk of bias,” “some concerns,” or “high risk of bias.” An overall assessment must be provided based on the RoB2 tool's algorithm.

Publication bias should be assessed using funnel plots and Egger's test, provided there are at least 10 studies available for direct comparison. The NMA should be conducted for both direct and indirect comparisons of interventions. Random-effects frequentist NMAs and pairwise meta-analyses should be used to assess transitivity and inform the strength of evidence. Coherence between direct and indirect evidence should be evaluated using the split node method.

Treatment rankings should be calculated based on the P score, an analog to the surface under the cumulative ranking (SUCRA), to determine the probability of each intervention being the most effective. These values can be used to rank treatments according to precision-weighted point estimates. All analyses must be performed using the netmeta package, version 2.8-0, in R (R Foundation for Statistical Computing).

### Ethics and Dissemination

The proposed systematic review follows the guidelines of the Brazilian National Health Council. As the review involves synthesizing previously published and anonymized data, it does not require formal review or approval from the Research Ethics Committee. A Declaration of Responsibility confirming this exemption is available upon request. The findings from this review will be disseminated through publication in peer-reviewed journals and presentations at relevant scientific conferences. The data will be made available upon reasonable request, following best practices in curation and deposition.

## Discussion

As a progressive fibroproliferative condition, DD significantly impacts hand functionality and quality of life. 5 26 Despite the availability of multiple treatments, such as percutaneous needle fasciotomy, fasciectomy, and CCH injections, the outcomes remain inconsistent, and recurrence rates are high. 6 27 The absence of standardized definitions for recurrence and the heterogeneity in outcome measures complicate direct comparisons between existing studies. 28 29 30 This protocol aims to address these gaps by employing a systematic approach to synthesize robust comparative evidence.

The present study adopts a PRISMA-based methodology to ensure transparency and reproducibility. 17 Key strengths include the application of NMA, which allows for both direct and indirect comparisons among multiple interventions, and the integration of planned subgroup analyses—considering variables such as patient age, comorbidities, and disease severity—to improve clinical applicability. Nonetheless, the review is expected to face challenges inherent to the literature, particularly the heterogeneity in outcome reporting and variability in study quality. 13 28 30 This variability poses a potential source of bias, which could affect the robustness of the planned analyses. These factors may introduce bias and affect the strength of the resulting evidence.

The need for a new systematic review on this topic is reinforced by the methodological limitations consistently observed in recent reviews. Most available studies have evaluated only two or three interventions, typically PNF, collagenase, and limited fasciectomy, thus excluding relevant options of treatment. Additionally, outcome reporting has been heterogeneous, with little to no use of structured PROM hierarchies. Most reviews have also conducted their literature searches over 5 years ago, thus failing to capture several high-quality randomized trials published more recently. 13 15 16 Collectively, these limitations highlight a gap in the literature that justifies the implementation of a comprehensive, up-to-date, and methodologically robust systematic review, as proposed in the present protocol.

The findings from the proposed review have the potential to inform treatment decisions and improve the clinical management of DD. By addressing critical gaps, especially the lack of standardized definitions and consistent outcome measures for functional improvement and adverse effects, this study may contribute to more uniform and reliable assessments in future research.

## Figures and Tables

**Table:** 

Search strategy for CENTRAL (via Cochrane Library) #1. MeSH descriptor: [Dupuytren Contracture] explode all trees #2. (Contracture, Dupuytren) OR (Dupuytren's Contracture) OR (Contracture, Dupuytren's) OR (Dupuytrens Contracture) OR (Fibromatosis, Palmar) OR (Palmar Fibromatosis) OR (Dupuytren's Disease) OR (Dupuytren Disease) OR (Dupuytrens Disease) #3. #1 AND #2 Search strategy for Medline (via PubMed) #1. “Dupuytren Contracture”[Mesh] OR (Contracture, Dupuytren) OR (Dupuytren's Contracture) OR (Contracture, Dupuytren's) OR (Dupuytrens Contracture) OR (Fibromatosis, Palmar) OR (Palmar Fibromatosis) OR (Dupuytren's Disease) OR (Dupuytren Disease) OR (Dupuytrens Disease) #2. (“clinical”[Title/Abstract] AND “trial”[Title/Abstract]) OR “clinical trials as topic”[MeSH Terms] OR “clinical trial”[Publication Type] OR “random*”[Title/Abstract] OR “random allocation”[MeSH Terms] OR “therapeutic use”[MeSH Subheading] #3. #1 AND #2 Search strategy for Embase (via Elsevier) #1. 'Dupuytren contracture'/exp OR 'contracture, dupuytren' OR 'contracture, palmar fascia' OR 'dupuytren disease' OR 'Dupuytreǹs contracture' OR 'palmar fascial contracture' OR 'palmar fibromatosis' OR 'Dupuytren contracture' #2. random* OR factorial* OR crossover* OR (cross over*) OR placebo* OR 'placebo'/exp OR (doubl* blind*) OR (singl* blind*) OR (assign* allocate*) OR volunteer* OR 'volunteer'/exp OR (crossover procedure) OR (double 'blind'/exp) procedure OR (randomized controlled trial) OR (single 'blind'/exp) OR procedure OR (controlled clinical trial) OR (clinical trial) #3. #1 AND #2 #4. #3 AND [embase]/lim NOT ([embase]/lim AND [medline]/lim) Search strategy for BVS Regional Portal #1. MH:”Contratura de Dupuytren” OR (Contratura de Dupuytren) OR (Dupuytren Contracture) OR (Contractura de Dupuytren) OR (Doença de Dupuytren) OR (Fibromatose Palmar) OR MH:C04.557.450.565.590.340.173$ OR MH:C05.651.197.270$ OR MH:C17.300.270$ #2. ((AB:ENSAIO$) OR (TW:ENSAIO$) OR (TI:ENSAIO$) OR (TRIAL$) OR (Ensayo$) OR (TW:Ensaio$ Clínico$ Controlado$) OR (AB:Ensaio$ Clínico$ Controlado$) OR (TI:Ensaio$ Clínico$ Controlado$) OR (ENSAIO$ CLINICO$) OR (Ensayo$ Clínico$) OR (Clinical Trial$) OR (ECR) OR (RCT) OR (ESTUDO$ RANDOMIZADO$ CONTROLADO$) OR (RANDOMISED CONTROLLED TRIAL$) OR (RANDOMIZADO$) OR (randomized controlled TRIAL) OR (QUASE RANDOMIZADO$) OR (ENSAIO$ QUASE RANDOMIZADO$) OR (ENSAIO$ CLINICO$ ALEATORIO$) OR (ENSAIO$ CLINICO$ CONTROLADO$) OR (RANDOMIZED SINGLE-BLIND CLINICAL TRIAL$) OR (RANDOMIZED SINGLE BLIND CLINICAL TRIAL$) OR (ensaio$ clínico$ randomizado$ simples cego) OR (CONTROL$) OR (RANDOM$) OR (PLACEBO$) OR (CUSTO) OR (EFICA$) OR (open controlled study) OR (TI:(Estudio doble ciego)) OR (ti:(Double-blind study)) OR (PT:”RANDOMIZED CONTROLLED TRIAL”) OR (“ENSAIO CLINICO CONTROLADO ALEATÓRIO”) OR (“ENSAYO CLINICO CONTROLADO ALEATORIO”) OR (“RANDOMIZED CONTROLLED CLINICAL TRIAL”) OR (“ENSAIO CONTROLADO ALEATÓRIO”) OR (“ENSAIO CLINICO CONTROLADO RANDOMIZADO”) OR (“ENSAYO CONTROLADO ALEATORIO”) OR (“ENSAYO CLINICO CONTROLADO RANDOMIZADO”) OR MH:V03.200.700$ OR PT:”CONTROLLED CLINICAL TRIAL” OR “ENSAIO CLINICO CONTROLADO” OR “ENSAYO CLINICO CONTROLADO” OR MH:V03.200.500$ OR MH:”RANDOMIZED CONTROLLED TRIALS AS TOPIC” OR “ENSAIOS CLINICOS CONTROLADOS ALAEATORIOS COMO ASSUNTO” OR “ENSAYOS CLINICOS CONTROLADOS ALEATORIOS COMO ASSUNTO” OR “CLINICAL TRIALS, RANDOMIZED” OR “ENSAIOS CLINICOS TERAPEUTICOS COMO ASSUNTO” OR “ENSAYOS CLINICOS CONTROLADOS COMO TEMA” OR MH:E05.318.760.535.365.500$ OR MH:E05.337.250.365.500$ OR MH:N05.715.360.775.235.387.500$ OR MH:N06.850.520.450.535.365.500$ OR MH:SP5.001.012.038.059.055$ OR MH:”RANDOM ALLOCATION” OR “DISTRIBUIÇÃO ALEATÓRIA” OR “DISTRIBUICION ALEATORIA” OR RANDOMIZATION OR “DISTRIBUIÇÃO CASUAL” OR RANDOMIZAÇÃO OR “ASIGNACION AL AZAR” OR RANDOMIZACION OR MH:E05.318.780.700$ OR MH:E05.581.500.805$ OR MH:N05.715.360.780.675$ OR MH:N06.850.520.445.700$ OR MH:”DOUBLE- BLIND METHOD” OR “DOUBLE-MASKED STUDY” OR “METODO DUPLO- CEGO” OR “METODO DOBLE CIEGO” OR “ESTUDIO DOBLE ENMASCARADO” OR MH:E05.318.780.300$ OR MH:E05.581.500.300$ OR MH:N05.715.360.780.320$ OR MH:N06.850.520.445.300$ OR MH:SP5.001.012.038.059.065$ OR MH:”SINGLE-BLIND METHOD” OR “METODO SIMPLES-CEGO” OR “METODO SIMPLE-CIEGO” OR “SINGLE- MASKED STUDY” OR “ESTUDIO SIMPLE ENMASCARADO” OR “METODO SIMPLECIEGO” OR MH:E05.318.780.850$ OR MH:N05.715.360.780.730$ OR MH:N06.850.520.445.850$ OR MH:SP5.001.012.038.059.070$ OR PT:”CLINICAL TRIAL” OR “ENSAIO CLINICO” OR “ENSAYO CLINICO” OR MH:V03.200$ OR MH:PLACEBOS OR PLACEBO$ OR MH:D26.660$ MH:E02.785$ or MH:HP3.073.433$ or MH:HP7.007.130$ OR MH:”RESEARCH DESIGN” OR “PROJETOS DE PESQUISA” OR “EXPERIMENTAL DESIGN” OR “PROYECTOS DE INVESTIGACION” OR “DESENHO EXPERIMENTAL” OR “PROYECTO EXPERIMENTAL” OR MH:E05.581.500$ OR MH:H01.770.644.728$ OR MH:SH1.020.010.070$ OR PT:”COMPARATIVE STUDY” OR “ESTUDO COMPARATIVO” OR “ESTUDIO COMPARATIVO” OR MH:V03.250$ OR PT:”EVALUATION STUDIES” OR “ESTUDOS DE AVALIAÇÃO” OR “ESTUDIOS DE EVALUACION” OR MH:V03.400$ OR PT:”FOLLOW-UP STUDIES” OR SEGUIMENTO$ OR “ESTUDIOS DE SEGUIMENTO” OR MH:E05.318.760.500.750.500.350$ OR MH:N05.715.360.775.175.250.350$ OR MH:N06.850.520.450.500.750.350$ OR MH:”PROSPECTIVE STUDIES” OR “ESTUDOS PROSPECTIVOS” OR “ESTUDIOS PROSPECTIVOS” OR MH:E05.318.760.500.750.500.650$ OR MH:N05.715.360.775.175.250.630$ OR MH:N06.850.520.450.500.750.650$) OR (Tw:clin$ AND (Tw:trial$ OR Tw:ensa$ OR Tw:estud$ OR Tw:experim$ OR Tw:investiga$)) OR ((Tw:singl$ OR Tw:simple$ OR Tw:doubl$ OR Tw:doble$ OR Tw:duplo$ OR Tw:trebl$ OR Tw:trip$) AND (Tw:blind$ OR Tw:cego$ OR Tw:ciego$ OR Tw:mask$ OR Tw:mascar$)) OR Mh:placebos OR Tw:placebo$ OR (Tw:random$ OR Tw:randon$ OR Tw:casual$ OR Tw:acaso$ OR Tw:azar OR Tw:aleator$) AND NOT (Ct:animal AND NOT (Ct:human and Ct:animal)) #3. #1 AND #2 Search strategy for Clinicaltrials.gov Dupuytren Contracture ICTRP search strategy Dupuytren Contracture

## Notes

Appendix 1 Search Strategies
